# Network meta‐analysis including treatment by covariate interactions: Consistency can vary across covariate values

**DOI:** 10.1002/jrsm.1257

**Published:** 2017-08-23

**Authors:** Sarah Donegan, Nicky J. Welton, Catrin Tudur Smith, Umberto D'Alessandro, Sofia Dias

**Affiliations:** ^1^ Department of Biostatistics University of Liverpool Waterhouse Building Liverpool UK; ^2^ School of Social and Community Medicine University of Bristol Bristol UK; ^3^ MRC Unit The Gambia Serrekunda The Gambia; ^4^ London School of Hygiene and Tropical Medicine London UK

**Keywords:** consistency, indirect comparison, individual patient data, meta‐regression, mnetwork meta‐analysis, treatment by covariate interaction

## Abstract

**Background:**

Many reviews aim to compare numerous treatments and report results stratified by subgroups (eg, by disease severity). In such cases, a network meta‐analysis model including treatment by covariate interactions can estimate the relative effects of all treatment pairings for each subgroup of patients. Two key assumptions underlie such models: consistency of treatment effects and consistency of the regression coefficients for the interactions. Consistency may differ depending on the covariate value at which consistency is assessed. For valid inference, we need to be confident of consistency for the relevant range of covariate values. In this paper, we demonstrate how to assess consistency of treatment effects from direct and indirect evidence at various covariate values.

**Methods:**

Consistency is assessed using visual inspection, inconsistency estimates, and probabilities. The method is applied to an individual patient dataset comparing artemisinin combination therapies for treating uncomplicated malaria in children using the covariate age.

**Results:**

The magnitude of the inconsistency appears to be decreasing with increasing age for each comparison. For one comparison, direct and indirect evidence differ for age 1 (P = .05), and this brings results for age 1 for all comparisons into question.

**Conclusion:**

When fitting models including interactions, the consistency of direct and indirect evidence must be assessed across the range of covariates included in the trials. Clinical inferences are only valid for covariate values for which results are consistent.

## 1 INTRODUCTION

When many treatments (eg, treatments *1*, *2*, and *3*) exist for the same condition and they form a connected network, network meta‐analysis (NMA) can estimate the relative effects of all treatment pairings (eg, *2* vs *1*, *3* vs *1*, and *3* vs *2*) using all direct and indirect evidence.^1^, ^2^, ^3^, ^4^, ^5^

The NMA models assume consistency between direct and indirect evidence for the treatment effects.^6^, ^7^ The assumption is satisfied when the consistency equations hold, for example, for a 3‐treatment network *d*_23_ = *d*_13_ − *d*_12_, where, for example, *d*_23_ is the treatment effect for *3* vs *2*. In other words, the assumption holds when, for each treatment pairing, the treatment effect is the same regardless of which trials allocated the 2 interventions. Methods to assess consistency include comparing characteristics, investigating treatment effect modifiers, comparing outcome measurements in the referent group, node splitting, inconsistency modelling, hypothesis tests, back‐transformation, multidimensional scaling, a 2‐stage approach, and a graph‐theoretical method.^3^, ^4^, ^6^, ^8^, ^9^, ^10^, ^11^, ^12^, ^13^, ^14^, ^15^

It is very common to explore treatment by covariate interactions in meta‐analyses using meta‐regression or subgroup analysis.^16^ Interactions can be included in an NMA model to evaluate whether each relative treatment effect varies with a covariate (eg, a patient or methodological characteristic, such as disease severity or allocation concealment).

The benefits of including interactions can be substantial. The model can produce the relative effects of all treatment pairings for each covariate value. For example, including an interaction for a categorical covariate, such as disease severity, which has 2 categories (ie, severe and nonsevere), would give one set of the relative effects for patients with severe disease and another set for patients with nonsevere disease. Similarly, using a continuous covariate (eg, patient age in years), the relative effects of all treatment pairings could potentially be calculated for any covariate value (eg, ages 1, 2, and 3). The estimation of results for each covariate value allows different recommendations to be made for different subgroups of patients; personalising treatment in this way can benefit patients and ensure the cost‐effective use of health care.^17^, ^18^, ^19^, ^20^, ^21^, ^22^, ^23^, ^24^, ^25^, ^26^ For example, as shown in an NMA, for the treatment of epilepsy, sodium valproate is recommended for patients with generalised seizures whereas carbamazepine is advised for patients with partial seizures.^26^, ^27^ Furthermore, when heterogeneity and/or inconsistency is detected in the NMA without interactions, results may be unreliable. If the treatment effect–modifying covariates that are causing the variability can be identified, results from models including interactions can be used to draw clinically meaningful results.

When fitting NMA models including interactions, we assume consistency of the treatment effects, where the treatment effects are estimated at the point where the covariate is zero (eg, *d*_23_ = *d*_13_ − *d*_12_) and consistency is also assumed for the regression coefficients for the interactions. As an example, for a 3‐treatment network, β_23_ = β_13_ − β_12_, where, for instance, β_23_ is the interaction regression coefficient for *3* vs *2*.^17^, ^18^, ^20^ The assumption for the coefficients holds when, for each treatment pairing, the coefficient is the same no matter which trials allocated the 2 interventions. Another way of viewing these 2 assumptions is simply that the treatment effects must be consistent at every covariate value. If such assumptions do not hold, results may be invalid and unreliable conclusions may be drawn.

**Figure 1:**
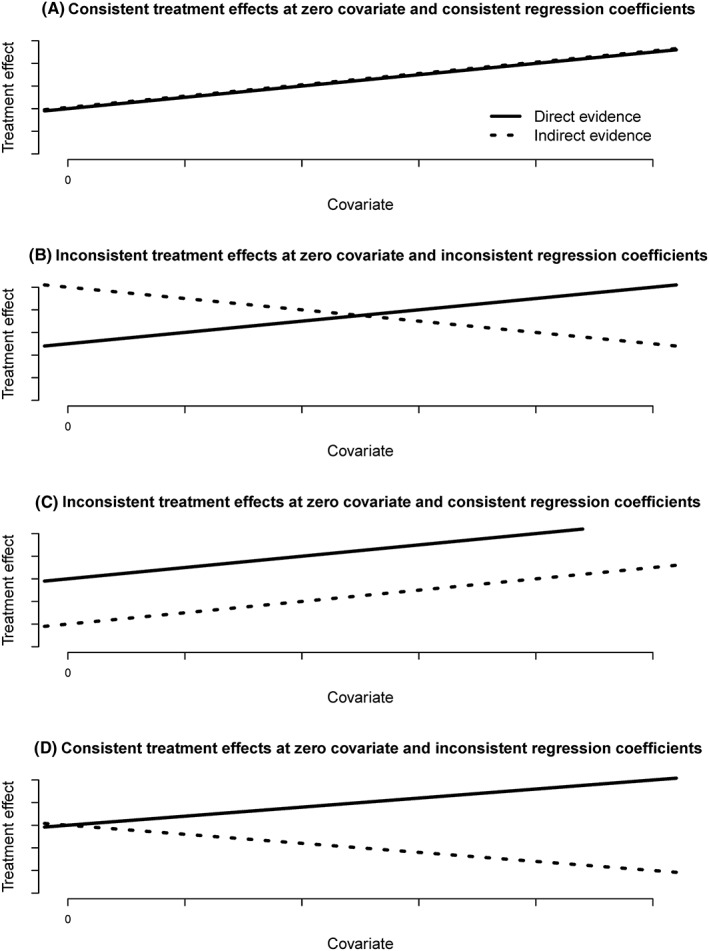
Graphs showing how the treatment effect for treatment *3* vs treatment *2* could change with a covariate value with separate lines representing direct evidence (from trials that allocated treatments *2* and *3*) and indirect evidence (from the remaining trials) when (A) the treatment effects at zero covariate are consistent and the regression coefficients for the treatment by covariate interaction are consistent; (B) the treatment effects at zero covariate are inconsistent and the coefficients are inconsistent; (C) the treatment effects at zero covariate are inconsistent and the coefficients are consistent; and (D) the treatment effects at zero covariate are consistent and the coefficients are inconsistent

Therefore, there are 4 possible scenarios that can occur when including interactions: both assumptions hold (ie, consistent treatment effects at the zero covariate value and consistent coefficients); neither assumption holds (ie, inconsistent treatment effects at zero covariate and inconsistent coefficients); or only one assumption holds (either consistency of the treatment effects at zero covariate or consistency of the coefficients). Figure 1A‐D shows examples of the 4 scenarios. The figures show how the treatment effect for *3* vs *2* changes with an increasing covariate value; separate regression lines are shown for direct and indirect evidence. The direct evidence for *3* vs *2* is from trials that allocated treatments *2* and *3*, and the indirect evidence for *3* vs *2* would be from the remaining trials. Note that the 2 lines have the same intercept when the treatment effects at the zero covariate value are consistent (Figure 1A and 1D) and the lines have the same slope when the coefficients are consistent (Figure 1A and 1C).

In Figure 1, the consistency of the treatment effects at each covariate value is represented by the distance between the 2 lines at each covariate value. We see that when both assumptions hold, the consistency of the treatment effects is the same regardless of the covariate value (Figure 1A). In Figure 1C, where there are consistent coefficients but inconsistent treatment effects at the covariate zero, the level of consistency of the treatment effects is the same at every covariate value. Yet in Figure 1B and 1D, where the coefficients are inconsistent, consistency of the treatment effects may differ depending on the covariate value at which consistency is assessed; at some values, there is consistency while at other values there is not. Notice that Figure 1B and 1D is essentially the same graph but with the y‐axis drawn at a different covariate value. In Figure 1D, there is consistency at the zero covariate, and in Figure 1B, there is consistency at a different point. For Figure 1B, if consistency is only assessed for the parameters estimated by the model (ie, the log odds ratios at zero covariate and the regression coefficient), the covariate range where there is reasonable consistency would be missed, whereas in the situation presented by Figure 1D, no points of consistency would be missed if only the parameters estimated by the model are assessed for consistency.

To draw reliable inferences, we must be confident that there is no evidence of inconsistency for the relevant range of covariates. Therefore, to gain a full understanding of consistency for a particular dataset, it is important to assess consistency at different covariate values to determine whether the consistency assumptions hold across the entire covariate range of interest, a limited covariate range, or not at all.

In this article, we demonstrate how to check consistency at different covariate values. To our knowledge, no other literature has highlighted this issue or demonstrated methods. We describe and demonstrate how consistency can change using real individual patient data with a dichotomous outcome and a continuous covariate under a Bayesian framework. However, the methods introduced certainly apply whenever NMA models exploring interactions are used including frequentist or Bayesian approaches and any data types.

## 2 METHODS

Here, we explain how to assess consistency at different covariate values by suggesting how to choose the covariate values at which to make the assessment, various ways to assess consistency, and possible conclusions to draw from the results.

### 2.1 Choosing covariate values

For continuous covariates, consistency can be assessed at particular covariate values that span the whole covariate range for which data are available. For example, for the covariate age in years, if the age of included patients in the trials ranged from 18 to 77 years, consistency could be assessed at equally spaced time points (eg, years 20, 30, 40, 50, 60, and 70). Of course, it is also important to consider whether results for particular covariate values are of interest for clinical decision‐making.

For categorical covariates, consistency can be assessed at each covariate value. For example, for the covariate disease severity, consistency could be assessed for the treatment effect applicable for patients with severe disease and then for those with nonsevere disease.

### 2.2 Ways to assess consistency

We propose 3 ways to assess consistency: visual inspection, calculating inconsistency estimates, and the corresponding probabilities of consistency.

To set notation, suppose we are comparing the direct and indirect evidence for a particular comparison, denoted as treatment *Z* vs treatment *Y*. For a treatment pair *YZ*, direct evidence would be from trials that allocated treatments *Y* and *Z*, whereas indirect evidence would be from the remaining trials.

Using direct evidence, suppose the treatment effect for *Z* vs *Y* at the covariate value zero is $d_{\mathrm{YZ}}^{\mathrm{dir}}$ and the regression coefficient for the treatment by covariate interaction for *Z* vs *Y* is $\beta_{\mathrm{YZ}}^{\mathrm{dir}}$. Therefore, the treatment effect for *Z* vs *Y* at covariate value *X* from direct evidence is given by

(1) $$d_{\mathrm{YZ}}^{\mathrm{dir}}+\beta_{\mathrm{YZ}}^{\mathrm{dir}}X.$$

Also, using indirect evidence, suppose $d_{\mathrm{YZ}}^{\mathrm{ind}}$ represents the treatment effect for *Z* vs *Y* at the covariate value zero and $\beta_{\mathrm{YZ}}^{\mathrm{ind}}$ represents the regression coefficient for *Z* vs *Y*. Then, the treatment effect for *Z* vs *Y* at covariate value *X* from indirect evidence is

(2) $$d_{\mathrm{YZ}}^{\mathrm{ind}}+\beta_{\mathrm{YZ}}^{\mathrm{ind}}X.$$

For a 3‐treatment network, estimates of the treatment effects at the covariate value zero (ie, $d_{12}^{\mathrm{dir}},$ $d_{13}^{\mathrm{dir}},\mathrm{and}\;d_{23}^{\mathrm{dir}}$) and of the regression coefficients (ie, $\beta_{12}^{\mathrm{dir}},$ $\beta_{13}^{\mathrm{dir}},\mathrm{and}\;\beta_{23}^{\mathrm{dir}}$) from direct evidence can be calculated by fitting multiple pairwise meta‐regression models. The corresponding results based on indirect evidence (ie, $d_{12}^{\mathrm{ind}},$ $d_{13}^{\mathrm{ind}},d_{23}^{\mathrm{ind}},\beta_{12}^{\mathrm{ind}},$ $\beta_{13}^{\mathrm{ind}},\mathrm{and}\;\beta_{23}^{\mathrm{ind}}$) could be calculated using the consistency equations, for example, $d_{23}^{\mathrm{ind}}=d_{13}^{\mathrm{dir}}-d_{12}^{\mathrm{dir}}$ and $\beta_{23}^{\mathrm{ind}}=\beta_{13}^{\mathrm{dir}}-\beta_{12}^{\mathrm{dir}}$. Then, for each chosen covariate value, the calculated estimates of the treatment effect and coefficients can be substituted into Equations (1) and (2) along with the covariate value to provide, for each covariate value, an estimate of the treatment effect for each comparison *Z* vs *Y* based on direct evidence and also estimates based on indirect evidence.

For larger networks, for each comparison *Z* vs *Y*, estimates of the treatment effects and regression coefficients based on direct evidence and estimates from indirect evidence can be calculated using more advanced techniques, such as node splitting or back‐calculation.^10^ Once these results have been obtained, consistency can then be assessed.

#### 2.2.1 Visual inspection

For each treatment comparison *Z* vs *Y*, at each covariate value *X*, consistency can be assessed by visually comparing the direction, size, and precision of the treatment effect estimated using indirect evidence (ie, $d_{\mathrm{YZ}}^{\mathrm{ind}}+\beta_{\mathrm{YZ}}^{\mathrm{ind}}X$) and that estimated from direct evidence (ie, $d_{\mathrm{YZ}}^{\mathrm{dir}}+\beta_{\mathrm{YZ}}^{\mathrm{dir}}X$).

#### 2.2.2 Inconsistency estimates

Also, for each comparison *Z* vs *Y*, at each covariate value, an inconsistency estimate can be calculated as the difference between the treatment effect estimated using indirect evidence (ie, $d_{\mathrm{YZ}}^{\mathrm{ind}}+\beta_{\mathrm{YZ}}^{\mathrm{ind}}X$) and that estimated from direct evidence (ie, $d_{\mathrm{YZ}}^{\mathrm{dir}}+\beta_{\mathrm{YZ}}^{\mathrm{dir}}X$). Therefore, the inconsistency estimate *w*_*YZ*_ is

$$w_{\mathrm{YZ}}=\left(d_{\mathrm{YZ}}^{\mathrm{dir}}+\beta_{\mathrm{YZ}}^{\mathrm{dir}}X\right)-\left(d_{\mathrm{YZ}}^{\mathrm{ind}}+\beta_{\mathrm{YZ}}^{\mathrm{ind}}X\right),$$

which can be rewritten as

(3) $$w_{\mathrm{YZ}}=\left(d_{\mathrm{YZ}}^{\mathrm{dir}}-d_{\mathrm{YZ}}^{\mathrm{ind}}\right)+X\left(\beta_{\mathrm{YZ}}^{\mathrm{dir}}-\beta_{\mathrm{YZ}}^{\mathrm{ind}}\right).$$

Large positive and large negative values of the inconsistency estimate would indicate inconsistency, whereas values near to zero would suggest agreement between direct and indirect evidence.

The inconsistency estimate can be calculated at different covariate values by substituting the estimated treatment effects and coefficients into Equation (3) along with the various chosen covariate values.

Notice that if the regression coefficient from direct evidence (ie, $\beta_{\mathrm{YZ}}^{\mathrm{dir}}$) is consistent with the regression coefficient from indirect evidence (ie, $\beta_{\mathrm{YZ}}^{\mathrm{ind}})$, that is,

$$\left(\beta_{\mathrm{YZ}}^{\mathrm{dir}}-\beta_{\mathrm{YZ}}^{\mathrm{ind}}\right)=0,$$

then the inconsistency estimate becomes

$$w_{\mathrm{YZ}}=\left(d_{\mathrm{YZ}}^{\mathrm{dir}}-d_{\mathrm{YZ}}^{\mathrm{ind}}\right),$$

and therefore, the estimate does not depend on the covariate value *X* and is the same for any covariate value. This is why the level of consistency is the same at every covariate value in Figure 1C.

However, if instead the treatment effect at the zero covariate value from direct evidence, $d_{\mathrm{YZ}}^{\mathrm{dir}}$, is consistent with that from indirect evidence (ie, $d_{\mathrm{YZ}}^{\mathrm{ind}})$, such that

$$\left(d_{\mathrm{YZ}}^{\mathrm{dir}}-d_{\mathrm{YZ}}^{\mathrm{ind}}\right)=0,$$

then the inconsistency estimate can be written as

$$w_{\mathrm{YZ}}=X\left(\beta_{\mathrm{YZ}}^{\mathrm{dir}}-\beta_{\mathrm{YZ}}^{\mathrm{ind}}\right),$$

and therefore the estimate is zero when the covariate is zero or when the regression coefficients from direct and indirect evidence are consistent (ie, $\left(\beta_{\mathrm{YZ}}^{\mathrm{dir}}-\beta_{\mathrm{YZ}}^{\mathrm{ind}}\right)=0$), and would depend on the covariate otherwise. This is the scenario observed in Figure 1D.

Yet if the treatment effects at the zero covariate value from direct and indirect evidence are inconsistent, that is,

$$\left(d_{\mathrm{YZ}}^{\mathrm{dir}}-d_{\mathrm{YZ}}^{\mathrm{ind}}\right)\neq 0,$$

and if

$$\left(\beta_{\mathrm{YZ}}^{\mathrm{dir}}-\beta_{\mathrm{YZ}}^{\mathrm{ind}}\right)>0,$$

then the inconsistency estimate increases with increasing covariate values, whereas if

$$\left(\beta_{\mathrm{YZ}}^{\mathrm{dir}}-\beta_{\mathrm{YZ}}^{\mathrm{ind}}\right)<0,$$

then the inconsistency estimate decreases with increasing covariate values. This scenario was presented in Figure 1B.

#### 2.2.3 Probabilities and hypothesis testing

Furthermore, to assess consistency, for each comparison *Z* vs *Y*, at each covariate value *X*, a Bayesian probability (eg, from node‐splitting models) can be calculated to determine the probability that the direct and indirect evidence agrees.^6^, ^10^ Further details are given in Section 3.2.

Under a frequentist approach (eg, using back‐calculation), for each comparison *Z* vs *Y*, at each covariate value *X*, a 2‐sample *t* test can be performed provided no multiarm trials contribute to the evidence, to test the null hypothesis that there is no difference between direct and indirect evidence.^10^ The inconsistency estimate

$$w_{\mathrm{YZ}}=\left(d_{\mathrm{YZ}}^{\mathrm{dir}}+\beta_{\mathrm{YZ}}^{\mathrm{dir}}X\right)-\left(d_{\mathrm{YZ}}^{\mathrm{ind}}+\beta_{\mathrm{YZ}}^{\mathrm{ind}}X\right)$$

has variance

$$\mathrm{var}\;\left(w_{\mathrm{YZ}}\right)=\mathrm{var}\left(d_{\mathrm{YZ}}^{\mathrm{dir}}+\beta_{\mathrm{YZ}}^{\mathrm{dir}}X\;\right)+\mathrm{var}\left(d_{\mathrm{YZ}}^{\mathrm{ind}}+\beta_{\mathrm{YZ}}^{\mathrm{ind}}X\right).$$

The test statistic

$$T_{\mathrm{YZ}}=\frac{w_{\mathrm{YZ}}}{\sqrt{\mathrm{var}\;\left(w_{\mathrm{YZ}}\right)}}$$

can be compared with the standard *t* distribution to obtain a *P* value. Small *P* values indicate a significant difference between the results from direct and indirect evidence. Previously, similar consistency tests have used a level of 5%^14^, ^28^, ^29^, ^30^ or 10%^29^, ^30^, ^31^ to denote statistical significance; we advocate using the 10% level because it errs on the side of caution.

### 2.3 Interpreting results

For continuous covariates, if the direct and indirect evidence appears consistent across the whole covariate range studied, the results from the NMA model including interactions are valid for that covariate range. Conversely, if the direct and indirect evidence is inconsistent across the whole covariate range studied, the results from the model are invalid for that covariate range. If the direct and indirect evidence is consistent for some covariate values studied and inconsistent at other covariate values, the results from the model would be useable for the covariate range where consistency is observed.

Similarly, for categorical covariates, the results from the model are valid if the direct and indirect evidence is consistent for each category studied; the results from the model are not reliable if the evidence is inconsistent for each category studied; and it may be appropriate to draw conclusions from the model for the categories where consistency is observed if the evidence is consistent for some categories studied and inconsistent for other categories.

## 3 ILLUSTRATIVE APPLICATION

In this section, the described methods are demonstrated through application to an individual patient dataset.

### 3.1 The dataset

The individual patient data are from a trial performed at sites across Africa that randomised children with uncomplicated *Plasmodium falciparum* malaria.^32^ Four artemisinin‐based combination therapies were compared: amodiaquine‐artesunate (AQ + AS), dihydroartemisinin‐piperaquine (DHAPQ), artemether‐lumefantrine (AL), and chlorproguanil‐dapsone plus artesunate (CD + A). Meta‐analysis was used to analyse the trial because investigators at each site chose which treatments they wanted to allocate (on the basis of antimalarial resistance and malaria endemicity); this is analogous to a meta‐analysis of trials where trial investigators choose treatments to randomise in their study.

Treatment success at day 28 was an outcome, and patient age was considered to be a potentially treatment effect–modifying covariate. The data consisted of the treatment allocation, outcome, site, and baseline age, of each child. Table S1 displays a summary of the data.

**Figure 2:**
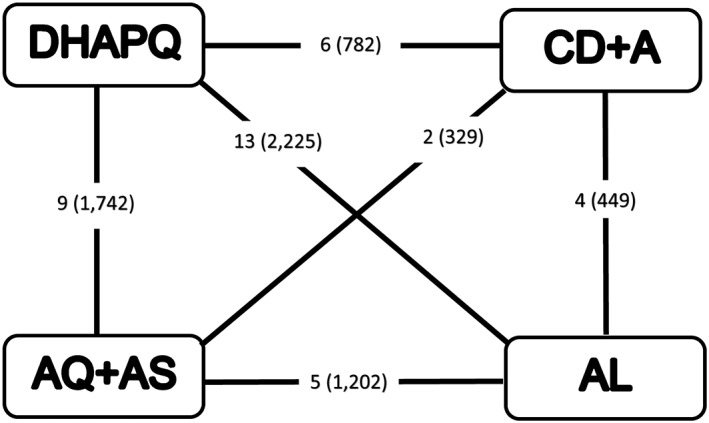
Network diagram of artemisinin‐based combination therapies. AL indicates artemether‐lumefantrine; AQ + AS, amodiaquine‐artesunate; CD + A, chlorproguanil‐dapsone plus artesunate; DHAPQ, dihydroartemisinin‐piperaquine. Number of sites (number of patients) displayed

The 17 sites each included 2 or 3 of the following treatments: DHAPQ, AQ + AS, AL, and CD + A. All 6 comparisons were supported by direct evidence (Figure 2).

### 3.2 Implementation

We fitted a standard NMA model including a treatment by covariate interactions and a second node‐splitting NMA model that provided estimates of the treatment effect at zero covariate and estimates of the coefficient based on direct evidence alone and separate estimates from indirect evidence alone. Correlation between treatment effects from the same site was taken into account in the models. In both models, we fixed the between‐site variances to be identical across treatment comparisons. See the Supporting Information for modelling specifications and code.

The drugs were ordered by treatment success rate: DHAPQ (1), AQ + AS (2), AL (3), and CD + A (4). The covariate was centred at its mean. In a node‐splitting model, direct and indirect evidence is separated for 1 comparison. Node splitting could not be used to assess consistency for AQ + AS vs DHAPQ because of the nature of the multiarm trials in the network, which meant that no indirect evidence contributed to the comparison AQ + AS vs DHAPQ after direct evidence was removed from the network. Similarly, node‐splitting models could not be used for AL vs DHAPQ or CD + A vs DHAPQ.

WinBUGS 1.4.3 and the R2WinBUGS package in R were used to fit the models.^33^, ^34^, ^35^ A uniform prior distribution (ie, σ ~ uniform (0, 10)) was chosen for the between‐site standard deviation. All other parameters were given noninformative normal prior distributions (ie, normal (0, 100 000)). Three chains with different initial values were run for 300 000 iterations. The initial 100 000 draws were discarded, and chains were thinned such that every fifth iteration was retained. Convergence was assessed using Gelman–Rubin plots, trace plots, and autocorrelation plots.

Log odds ratios for children aged 1, 2, 3, 4, and 5 years were reported. These covariate values were chosen because the age of children included in the trial ranged from 6 months to 5 years, and age is traditionally expressed by year. Also, results for children of average age were presented because the models were centred at the mean.

The inconsistency estimate was calculated at each iteration of the chain. A probability was estimated by counting the number of iterations for which the estimate was positive (≥0) and then calculating the probability (ie, prob) that the estimate was positive, by dividing the number of counted iterations by the total number of iterations of the chain. The probability value corresponding with a 2‐tailed test was obtained by *P* = 2 × minimum (prob, 1 − prob) that represents the probability of consistent direct and indirect evidence with lower probabilities indicating lower levels of agreement.^10^, ^36^ The posterior distribution of the inconsistency estimates was checked for symmetry and unimodality.

## 4 RESULTS

### 4.1 Visual inspection

**Figure 3:**
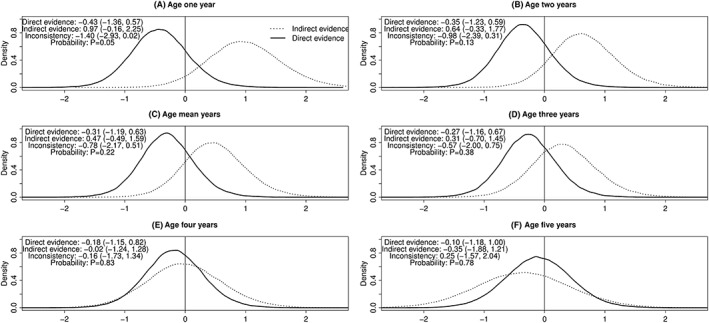
A‐F, Posterior distributions of log odds ratios at various ages for treatment success for AL versus AQ + AS. AL indicates artemether‐lumefantrine; AQ + AS, amodiaquine‐artesunate. The mean age was 2.5 years. Posterior median (95% credibility interval) presented

**Figure 4:**
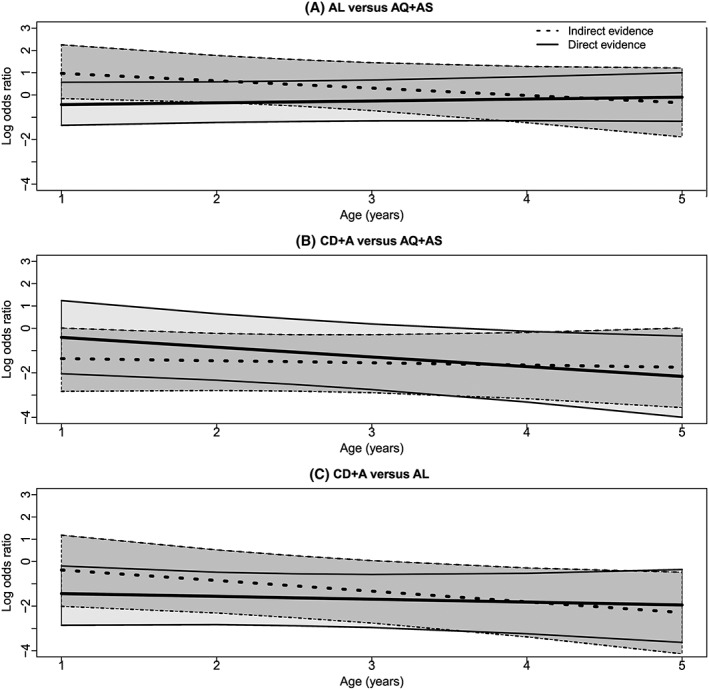
A‐C, Graphs showing, for each comparison, how the posterior median of the log odds ratio for treatment success and its 95% credibility interval change with age; separate results are presented for direct evidence and indirect evidence. Lines represent the posterior median and upper and lower bound of the 95% credibility interval, and the shaded area represents the 95% credibility interval. AL indicates artemether‐lumefantrine; AQ + AS, amodiaquine‐artesunate; CD + A, chlorproguanil‐dapsone plus artesunate; DHAPQ, dihydroartemisinin‐piperaquine

For AL vs AQ + AS, Figure 3 displays, for each age, the posterior distribution of the log odds ratio from direct evidence and indirect evidence. At each age, the direct and indirect evidence differs in terms of the direction and size of the log odds ratio, but not the precision. The magnitude of the inconsistency appears to be decreasing with increasing age. Table S2 displays the corresponding odds ratios, which also differ in terms of size and direction and also precision. Figure 4 shows how age varies the log odds ratio. For AL vs AQ + AS, there are differences between direct and indirect evidence for both the intercept and slope; in particular, the log odds ratio increases with age using direct evidence, and it decreases with age using indirect evidence.

For CD + A vs AQ + AS, the results from direct and indirect evidence differ but not as much as for AL vs AQ + AS (Figure S1). Differences exist between direct and indirect evidence with respect to the size of the log odds ratio. The magnitude of the inconsistency estimate decreases with increasing age. Differences are also seen between odds ratios from direct and indirect evidence (Table S2). In Figure 4, differences exist between direct and indirect evidence for the slope and intercept for CD + A versus AQ + AS. However, in this case, the log odds ratio decreases with age regardless of the type of evidence used.

Similarly, for CD + A vs AL, the results from direct and indirect evidence are quite similar in terms of the precision but not the size of the log odds ratio (Figure S2). Also, the magnitude of the inconsistency decreases with increasing age. Table S2 shows the odds ratios from direct and indirect evidence also differ slightly in terms of size. The intercept and slope estimated using direct evidence differ from those using indirect evidence; but the log odds ratio decreases with age for each evidence type (Figure 4).

### 4.2 Inconsistency estimates

**Figure 5:**
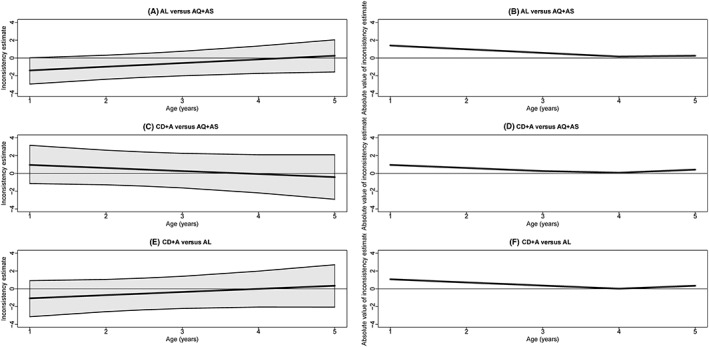
Graphs showing, for each comparison, how the posterior median of the inconsistency estimate and its 95% credibility interval change with age (A, C, and E) and how the absolute value of the posterior mean of the inconsistency estimate changes with age (B, D, and F). AL indicates artemether‐lumefantrine; AQ + AS, amodiaquine‐artesunate; CD + A, chlorproguanil‐dapsone plus artesunate; DHAPQ, dihydroartemisinin‐piperaquine

Figure 5 displays how the inconsistency estimate changes with the covariate. For AL vs AQ + AS, the inconsistency estimate increases with increasing age (from −1.40 to 0.25); yet the absolute value of the estimate decreases from 1.40 for age 1 to 0.16 for age 4 and then increases to 0.25 for age 5. For CD + A vs AQ + AS, the inconsistency estimate decreases with increasing age (from 0.95 to −0.42), but the absolute value of the estimate decreases from 0.95 for age 1 to 0.07 for age 4 and then increases to 0.42 for age 5. For CD + A vs AL, the inconsistency estimate increases with age (from −1.07 to 0.34), while the absolute value of the estimate decreases from 1.07 for age 1 to 0.01 for age 4 and then increases to 0.34 for age 5.

### 4.3 Probabilities

For AL vs AQ + AS, there is a low level of agreement between direct and indirect evidence for age 1 (*P* = .05) (Figure 3). For CD + A vs AQ + AS and CD + A vs AL, the probabilities indicate reasonable agreement between direct and indirect evidence (Figures S1 and S2).

### 4.4 Overall interpretation

The magnitude of the inconsistency appears to be decreasing with increasing age for each comparison. Using the probabilities, there is inconsistency around age 1 year for AL vs AQ + AS, and arguably, the results are reasonably consistent for ages 2 to 5 years for all comparisons; therefore, the results from the NMA model including interactions could be used to draw inferences for children aged 2 to 5 years. Further results from the model are shown in Table S3.

## 5 DISCUSSION

We have demonstrated that, when fitting NMA models including interactions, the level of consistency of direct and indirect evidence can change with the covariate value. We have shown that it is important to check consistency at different values and have suggested how to do this.

When we applied the method, we found inconsistent evidence for AL vs AQ + AS at age 1 (*P* = .05). If we had only assessed consistency at a particular covariate value (eg, at the mean age), we could have incorrectly concluded that the results were consistent at any covariate value and drawn unreliable conclusions. This shows that consistency must be assessed at a range of covariate values, rather than one particular value.

Using the probabilities, we found that the log odds ratios for all comparisons were consistent for ages 2 to 5 years; therefore, the results from the model including interactions could be used to draw inferences for this age range. However, an inconsistency is observed at age 1 for AL vs AQ + AS, and this brings results at age 1 for all comparisons into question because we do not know whether the direct and/or indirect evidence is unreliable.^10^ Therefore, it may be more appropriate to reconsider all the evidence by exploring other potential treatment effect–modifying covariates, considering nonlinear relationships and checking methodological rigour and risk of bias of the evidence sources.

One possible explanation for the inconsistency at age 1 for AL vs AQ + AS is that the eligibility criteria in terms of age differed across sites. The direct evidence is from 5 sites where children aged 6 to 59 months were eligible. However, the indirect evidence is based on 6 sites where children aged 6 to 59 months were eligible (2 sites compared AQ + AS vs DHAPQ and 4 sites compared AL vs DHAPQ) and six sites where children aged 12 to 59 months were eligible (2 sites compared AQ + AS vs DHAPQ and 4 sites compared AL vs DHAPQ).

The methods presented in this article are extremely flexible. We applied the methods to individual patient data for a dichotomous outcome and continuous covariate using Bayesian methods, but they can be easily adapted to accommodate aggregated data, other outcome and covariate data types, and frequentist methods.

The models used in the application assumed that the regression coefficients for each treatment versus the reference treatment are independent. However, other model specifications are possible; for instance, exchangeable interactions assume that the regression coefficients are related and follow a distribution, and common interactions assume the coefficients are the same.^17^, ^18^, ^20^ The methods presented in this article can be applied with different modelling specifications.

We have applied similar models to aggregate datasets, and application is straightforward (application not presented in this article). However, if aggregate data are limited, it can be difficult to identify inconsistencies if they exist because credibility intervals can be wide. When using aggregate data, if for a particular comparison only one study contributes direct evidence, the regression coefficient based on direct evidence would be based on prior information (using a Bayesian approach) or would not be estimable (using a frequentist approach). To overcome the problem, exchangeability of coefficients could be assumed to borrow strength from other coefficients, or informative prior distributions may be used. In any case, node‐splitting models may be applied for other comparisons to assess consistency.

In this article, consistency was assessed using node splitting. Node‐splitting models have the advantage of providing an estimate based on direct evidence and an estimate from indirect evidence along with agreement probabilities, and they can take the correlation in multiarm trials into account. However, other methods, for example, back‐calculation, may be used to assess consistency in NMA models including interactions. Regardless of the method chosen, consistency across various covariate values must be considered.

The main limitation of this research is that the methods have only been demonstrated using one dataset; therefore, the methodology should certainly be applied in other contexts to further evaluate the strengths and weaknesses of the method. As with all NMA methods, we anticipate that application of the methods to large networks will become more complicated to apply and report. In particular, in such cases, the number of possible comparisons where consistency can be assessed can be large and therefore time‐consuming to assess. There are automatic routines, which are particularly useful for complex networks, to identify the relevant comparisons for a given network.^37^

The presented methods assume that within‐trial interactions and across‐trial interactions are equivalent. If ecological bias is at play, across‐trial information may be biased and may differ from interactions found within trials.^38^, ^39^ The NMA models that separate within‐trial and across‐trial interaction have been previously proposed to explore biases.^19^, ^20^ The principles presented in this article could be extended to accommodate within‐trial and across‐trial information.

Furthermore, multiple testing issues can play a role especially for large networks. When many statistical tests are performed, there is an increased chance of incorrectly concluding there is inconsistency when, in fact, results are consistent. At any rate, detecting inconsistency when there is no real inconsistency is erring on the side of caution. Assessments of consistency should be based on visual inspection as well as probabilities or *P* values. Methods used to adjust for multiple testing in a frequentist framework (eg, Bonferroni corrections) could be used to aid interpretation of *P* values in both frequentist and Bayesian frameworks.

Lastly, extrapolation issues may arise. Extrapolation can occur when the covariate distribution differs across comparisons and the results are interpreted across the whole covariate range of the included studies. However, interpreting results across this covariates range allows one to make predictions for treatment effects at covariate values where there may be no evidence. Although this can be attractive, such predictions must be interpreted with caution.

In conclusion, it is important to evaluate the consistency of direct and indirect evidence at various covariate values when fitting models including interactions because the level of consistency can change with the covariate value. Clinical inferences may be drawn for covariate values for which results are consistent.

## FUNDING INFORMATION

This research was supported by the Medical Research Council (grant MR/K021435/1) as part of a career development award in biostatistics awarded to S.Do.

## CONFLICT OF INTEREST

The authors have declared that no competing interests exist.

## AUTHOR CONTRIBUTIONS

S.Do., S.Di., and N.W. proposed the idea of exploring consistency at different covariate values in a network meta‐analysis model that included treatment by covariate interactions. S.Do. performed the analysis and wrote the first draft of the manuscript. S.Di., C.T.S., and N.W. provided statistical guidance and commented on the manuscript. U.D. provided helpful discussions regarding clinical aspects.

## Supporting information

Table S1. Summary of the individual patient data (i.e. event rate of each treatment group of each site for treatment success at day 28) and covariate information.
Table S2. Odds ratios for treatment success from the NMA node‐splitting models including interactions (model S2).
AQ + AS: amodiaquine‐artesunate; AL: artemether‐lumefantrine; CD + A: chlorproguanil‐dapsone plus artesunate; DHAPQ: dihydroartemisinin‐piperaquine.
Table S3. Selected results for treatment success from the NMA model including interactions (model S1).
AQ + AS: amodiaquine‐artesunate; AL: artemether‐lumefantrine; CD + A: chlorproguanil‐dapsone plus artesunate; DHAPQ: dihydroartemisinin‐piperaquine.
The between trial variance was 0.77 (0.27, 2.07).
Figure S1. Posterior distributions of log odds ratios at various ages for treatment success for CD + A versus AQ + AS.
The mean age was 2.5 years.
AQ + AS: amodiaquine‐artesunate; CD + A: chlorproguanil‐dapsone plus artesunate.
Posterior median (95% credibility interval) presented.
Figure S2. Posterior distributions of log odds ratios at various ages for treatment success for CD + A versus AL.
The mean age was 2.5 years.
AL: artemether‐lumefantrine; CD + A: chlorproguanil‐dapsone plus artesunate.
Posterior median (95% credibility interval) presented.
