# Supplementary material for: Network meta‐analysis including treatment by covariate interactions: Consistency can vary across covariate values
Source: Res Synth Methods. 2017 Aug 23;8(4):485–95. doi: 10.1002/jrsm.1257 (PMC5724666; doi:10.1002/jrsm.1257)
Supplement: Supplementary file 1 — Table S1. Summary of the individual patient data (i.e. event rate of each treatment group of each site for treatment success at day 28) and covariate information. Table S2. Odds ratios for treatment success from the NMA node‐splitting models including interactions (model S2). AQ + AS: amodiaquine‐artesunate; AL: artemether‐lumefantrine; CD + A: chlorproguanil‐dapsone plus artesunate; DHAPQ: dihydroartemisinin‐piperaquine. Table S3. Selected results for treatment success from the NMA model including interactions (model S1). AQ + AS: amodiaquine‐artesunate; AL: artemether‐lumefantrine; CD + A: chlorproguanil‐dapsone plus artesunate; DHAPQ: dihydroartemisinin‐piperaquine. The between trial variance was 0.77 (0.27, 2.07). Figure S1. Posterior distributions of log odds ratios at various ages for treatment success for CD + A versus AQ + AS. The mean age was 2.5 years. AQ + AS: amodiaquine‐artesunate; CD + A: chlorproguanil‐dapsone plus artesunate. Posterior median (95% credibility interval) presented. Figure S2. Posterior distributions of log odds ratios at various ages for treatment success for CD + A versus AL. The mean age was 2.5 years. AL: artemether‐lumefantrine; CD + A: chlorproguanil‐dapsone plus artesunate. Posterior median (95% credibility interval) presented. [file JRSM-8-485-s001.pdf]

Supporting materials

Supplementary models

Details of the individual patient data network meta-analysis models including treatment by covariate interactions that were applied are given below.

Notation

Let i denote the trial where $i = 1, \ldots \ldots, NS$ and NS is the number of independent trials; let j the patient where $j = 1, \ldots \ldots, NP_i$ such that NP_i is the number of patients in trial i; and let k be the trial arm where $k = 1, \ldots \ldots, NA_i$ and NA_i is the number of arms in trial i.

Suppose $y_{ijk} = 1$ if patient j in trial i in arm k experiences the event and $y_{ijk} = 0$ if patient j in trial i in arm k does not experience the event. Assume that the outcomes of patients, y_{ijk}, are independent and distributed as $y_{ijk} \sim bernoulli(p_{ijk})$ where p_{ijk} is the probability of an event for patient j in trial i in arm k. Let x_{ijk} be a patient-level covariate for patient j in trial i in arm k (such as, a continuous covariate value or an indicator variable for a dichotomous covariate).

Let t_{ik} denote the treatment given in trial i in arm k where $t_{ik} \in \{1, \ldots \ldots, NT\}$ and NT is the number of treatments in the network. Also specify that the node being split is $(\hat{t},\ t^*)$ where $\hat{t} \neq\ t^*$ and $\hat{t} <\ t^*$. For example, if one wants to split the node (*3*, *4*) then $\hat{t} = 3$ and $t^* = 4$.

Model S1. NMA model including treatment by covariate interaction

Assuming no multi-arm trials exist, the random-effects model is given as follows:

$$logit(p_{jik}) = \begin{cases} \mu_i + \beta_{0i} x_{jik} & if\ k = 1 \\ \mu_i + \beta_{0i} x_{jik} + \delta_{i,\,1k} + \beta_{t_{i1},t_{ik}} x_{jik} & if\ k \neq 1 \end{cases}$$

where μ_i is the log odds of an event in arm *1* of trial *i*; β_{0i} is a study-specific regression parameter that represents the difference in the log odds of an event in arm *1* of trial *i* per unit increase in the covariate x_{ijk}; $\beta_{t_{i1},t_{ik}}$ represents the difference in the log odds ratio of t_{ik} vs. t_{i1} per unit increase in the covariate and $\beta_{t_{i1},t_{ik}}$=$\beta_{1,t_{ik}}$-$\beta_{1,t_{i1}}$; and $\delta_{i,\,1k}$ represents the trial-specific log odds ratio of t_{ik} vs. t_{i1}. The trial-specific log odds ratios, $\delta_{i,\,1k}$ are assumed to be realisations from a normal distribution where

$$\delta_{i,1k} \sim N(d_{t_{i1},t_{ik}},\ \sigma^2)$$

and

$$d_{t_{i1},t_{ik}} = d_{1,t_{ik}} - d_{1,t_{i1}}$$

In this model, $d_{t_{i1},t_{ik}}$ represents the log odds ratio of t_{ik} vs. t_{i1}. The fixed-effect model is given by setting $\sigma^2 = 0$.

Under a Bayesian framework, prior distributions are specified for μ_i, β_{0i}, $d_{1,t_{ik}}$, $\beta_{1,t_{ik}}$ and σ^2.

The model can also be applied to datasets with multi-arm trials but the correlation between trial-specific treatment effects must be taken into account. For each multi-arm trial *i* with *m*

arms, the trial-specific treatment effects are taken to be a realisation from a multivariate normal distribution

$$\begin{pmatrix} \delta_{i,\,12} \\ \vdots \\ \delta_{i,\,1m} \end{pmatrix} \sim N\left(\begin{pmatrix} d_{1,t_{i2}} - d_{1,t_{i1}} \\ \vdots \\ d_{1,t_{im}} - d_{1,t_{i1}} \end{pmatrix}, \begin{pmatrix} \tau^2 & \dots & \tau^2/2 \\ \vdots & \ddots & \vdots \\ \tau^2/2 & \dots & \tau^2 \end{pmatrix} \right)$$

that can be decomposed into a series of conditional univariate normal distributions.

Model S2. NMA node-splitting model including treatment by covariate interaction

When there are no multi-arm trials, the random-effects model is specified as follows:

$$logit(p_{ijk}) = \begin{cases} \mu_i + \beta_{0i} x_{ijk} & if\ k = 1 \\ \mu_i + \beta_{0i} x_{ijk} + \delta_{i,\,1k} + \beta_{t_{i1},t_{ik}} x_{ijk} & if\ k \neq 1\ and\ t_{i1} \neq \hat{t} \text{ and/or } t_{ik} \neq t^* \\ \mu_i + \beta_{0i} x_{ijk} + \delta_{i,\,1k} + \beta^{dir} x_{ijk} & if\ k \neq 1\ and\ t_{i1} = \hat{t} \text{ and } t_{ik} = t^* \end{cases}$$

and where β^{dir} represents the difference in the log odds ratio of t^* vs. $\hat{t}$ per unit increase in the covariate estimated using direct evidence; $\beta_{t_{i1},t_{ik}}$ represents the difference in the log odds ratio of t_{ik} vs. t_{i1} per unit increase in the covariate estimated using all trials that did not allocate $t^*$and $\hat{t}$ (i.e. using indirect evidence); and $\delta_{i,\,1k}$ represents the trial-specific log odds ratio of t_{ik} vs. t_{i1}. The trial-specific log odds ratios, $\delta_{i,\,1k}$ are assumed to be realisations from a normal distribution where

$$\delta_{i,1k} \sim N(d^{dir},\ \sigma^2)$$

if trial *i* allocated $t^*$and $\hat{t}$, that is, $t_{i1} = \hat{t}$ and $t_{ik} = t^*$; whereas

$$\delta_{i,1k} \sim N\left(d_{t_{i1},t_{ik}},\ \sigma^2\right)$$

and the treatment effects satisfy the consistency equation $d_{t_{i1},t_{ik}} = d_{1,t_{ik}} - d_{1,t_{i1}}$ if trial i did not allocate $t^*$and $\hat{t}$, that is, $t_{i1} \neq \hat{t}$ and/or $t_{ik} \neq t^*$.

In this model $d_{t_{i1},t_{ik}}$ represents the mean log odds ratio of t_{ik} vs. t_{i1} when the covariate value is zero estimated using all studies that did not allocate t^* and $\hat{t}$ (i.e. using indirect evidence); and d^{dir} represents the mean log odds ratio of t^*vs. $\hat{t}$ when the covariate value is zero estimated using direct evidence.

Under a Bayesian framework, prior distributions are specified for μ_i, β_{0i}, $d_{1,t_{ik}}$, $\beta_{1,t_{ik}}$, d^{dir}, β^{dir} and σ^2.

Multiple node-splitting models are usually applied. One model can be applied for each comparison providing both direct and indirect evidence are available for that comparison.

Node-splitting models can accommodate multi-arm trials as described elsewhere (Dias et al., 2010a, van Valkenhoef et al., 2016). If one wants to split node (t_{i1}, t_{ik}) then a multi-arm trial i will contribute direct evidence to the treatment effect (d^{dir}) because $\hat{t} = t_{i1}$. However, if one splits another node $(\text{e.g.}\,(t_{i2}, t_{i3}))$ then $\hat{t} \neq t_{i1}$ therefore, the multi-arm trial would not contribute direct evidence to the estimation of the treatment effect (d^{dir}), therefore, to overcome this problem and to utilise all the direct evidence, if the multi-arm trial compared the two treatments t^* and $\hat{t}$, in addition to other treatments, treatment $\hat{t}$ is taken to be the

baseline treatment t_{i1} for that study. For example, if a trial *i* compared treatments *1*, *3* and *4*, and one wants to split node (*1*, *3*) then $\hat{t} = t_{i1} = 1$ and the model would be as follows:

$logit(p_{ij1}) = \mu_i + \beta_{0i}x_{ij1}$ for treatment *1*,

$logit(p_{ij2}) = \mu_i + \beta_{0i}x_{ij2} + \delta_{i,\,12} + \beta^{dir}x_{ij2}$ for treatment *3* where $\delta_{i,\,12} \sim N\left(d^{dir}, \tau^2\right)$, and

$logit(p_{ij3}) = \mu_i + \beta_{0i}x_{ij3} + \delta_{i,\,13} + \beta_{1,4}x_{ij3}$ for treatment *4* where $\delta_{i,\,13} \sim N\left(d_{1,4}, \tau^2\right)$.

Whereas, for the same trial, if one wants to split node (*3*, *4*) instead, then we fix $\hat{t} = t_{i1} = 3$ and the model is

$logit(p_{ij2}) = \mu_i + \beta_{0i}x_{ij2} + \delta_{i,\,12} + \beta_{3,1}x_{ij2}$ for treatment *1* where $\delta_{i,\,12} \sim N\left(d_{3,1}, \tau^2\right)$.

$logit(p_{ij1}) = \mu_i + \beta_{0i}x_{ij1}$ for treatment *3*, and

$logit(p_{ij3}) = \mu_i + \beta_{0i}x_{ij3} + \delta_{i,\,13} + \beta^{dir}x_{ij3}$ for treatment *4* where $\delta_{i,\,13} \sim N\left(d^{dir}, \tau^2\right)$.

Code for Model S1

Winbugs code (saved as winbugs file "NMA RE IPD COVM1.odc")

```
model{
for(i in 1:ns){                                  #LOOP FOR EACH TRIAL
   w[i,1] <- 0                                   #W IS ZERO FOR ARM 1 OF EACH TRIAL
   delta[i,1] <- 0                               #TREATMENT EFFECT IS ZERO FOR ARM 1 OF EACH TRIAL
   mu[i] ~ dnorm(0,0.00001)                      #PRIOR DISTRIBUTION FOR MU
   beta0[i] ~ dnorm(0,0.00001)                   #PRIOR DISTRIBUTION FOR BETA0
      for (k in 2:na[i]) {                       #LOOP FOR EACH ARM
         delta[i,k] ~ dnorm(md[i,k], taud[i,k])  #DISTRIBUTION OF TRIAL-SPECIFIC TREATMENT EFFECTS
         md[i,k] <-  d[t[i,k]] - d[t[i,1]] + sw[i,k]   #MEAN OF DISTRIBUTION (CORRECTED FOR MULTI-ARM
TRIALS)
         taud[i,k] <- tau *2*(k-1)/k             #PRECISION OF DISTRIBUTION (CORRECTED FOR MULTI-ARM
TRIALS)
         w[i,k] <- (delta[i,k] - d[t[i,k]] + d[t[i,1]]) #ADJUSTMENTS FOR MULTI-ARM TRIALS
         sw[i,k] <- sum(w[i,1:k-1])/(k-1)        #ADJUSTMENTS FOR MULTI-ARM TRIALS
                       }
              }
for(l in 1:np)  {                                                              #LOOP FOR EACH PATIENT
  y[l]~dbern(p[l])                                                             #BERNOULLI LIKELIHOOD
  logit(p[l])<-mu[s[l]] + (beta0[s[l]]*(x[l]-mx)) + delta[s[l],arm[l]] + (beta[tipd[l]]-beta[b[l]]) * (x[l]-mx)
                                                                               #LINEAR PREDICTOR
  rhat[l] <- p[l]                                                              #MODEL PREDICTION
  dev[l] <- 2*(y[l] * (log(y[l]/rhat[l])) + (1-y[l]) * (log((1-y[l])/(1-rhat[l]))))    #DEVIANCE
                }

totresdev <- sum(dev[])                          #TOTAL RESIDUAL DEVIANCE

d[1]<-0                                          #LOG ODDS RATIO IS ZERO FOR REFERENT TREATMENT
beta[1] <- 0                                     #COEFFICIENT IS ZERO FOR REFERENT TREATMENT

sd ~ dunif(0,10)                                 #PRIOR DISTRIBUTION FOR BETWEEN TRIAL STANDARD
                                                   DEVIATION
tau <- pow(sd,-2)                                #BETWEEN TRIAL PRECISION
tausq <- sd*sd                                   #BETWEEN TRIAL VARIANCE

for (k in 2:nt){
   d[k] ~ dnorm(0,0.00001)                       #PRIOR DISTRIBUTIONS
   beta[k]~dnorm(0,0.00001)
              }

for (k in 1:nt){                                 #CALCULATE THE LOG ODDS RATIO FOR BASIC
                                                   PARAMETERS AT EACH COVARIATE VALUE
   for (j in 1:nz) {
      dz[j,k] <- d[k] - (beta[k])*(mx-z[j]) }
                  }

for (c in 1:(nt-1)){                             #CALCULATE, FOR EACH COMPARISON, THE
                                                   COEFFICIENT, ODDS RATIO AND LOG ODDS RATIO AT
                                                   MEAN COVARIATE VALUE.

    for (k in (c+1):nt)  {
          betas[c,k] <- beta[k] - beta[c]
          or[c,k] <- exp(d[k] - d[c])
          lor[c,k] <- (d[k]-d[c])
             for (j in 1:nz) {
                                                 #CALCULATE, FOR EACH COMPARISON, ODDS RATIO AND
                                                   LOG ODDS RATIO AT DIFFERENT COVARIATE VALUES

                orz[j,c,k] <- exp(dz[j,k] - dz[j,c])
                lorz[j,c,k] <- (dz[j,k]-dz[j,c])
                             }
                         }
                  }
}
```

Dataset 1 (saved a csv file"utf_ipdacc.csv")
#t1= treatment in arm 1, t2=treatment in arm 2, t3=treatment in arm 3.
#na=number of arms
#Note that each row represents one study and the studies are in the same order as in dataset 2.

t1	t2	t3	na
1	2	NA	2
1	2	NA	2
1	2	3	3
1	2	3	3
1	2	3	3
1	2	3	3
1	2	3	3
1	2	4	3
1	2	4	3
1	3	NA	2
1	3	NA	2
1	3	NA	2
1	3	NA	2
1	3	4	3
1	3	4	3
1	3	4	3
1	3	4	3

Dataset 2 (saved as csv file "utf_ipdacc2.csv")
(one row per patient)
#age=covariate
#y=binary IPD outcome
#tipd=treatment
#s=study
#b=baseline treatment in that study
#arm=study arm (i.e. 1, 2, 3)
#note that arm 1 of each study is the baseline treatment for that study.

age	y	tipd	s	b	arm
21	1	1	1	1	1
29	1	1	1	1	1
.	.	.	.	.	.
.	.	.	.	.	.

R code

```
#INSTALL R PACKAGES
library(R2WinBUGS)
library(coda)

#CHOOSE WORKING DIRECTORY
working.directory="c:\\dir"
setwd(working.directory)

#IMPORT DATA
dat1 = read.csv("utf_ipdacc.csv")
dat2 = read.csv("utf_ipdacc2.csv")

#DEFINE VARIABLES THAT NEED TO BE ENTERED INTO THE WINBUGS MODEL
na=dat1$na                                          #NUMBER OF ARMS IN EACH STUDY
t=cbind(dat1$t1,dat1$t2,dat1$t3, deparse.level = 0)     #TREATMENT NUMBER
s=dat2$s                                            #STUDY NUMBER
y=dat2$y                                            #OUTCOME
arm=dat2$arm                                        #STUDYARM
x=dat2$age/12                                       #COVARIATE VALUES
b=dat2$b                                            #BASELINE TREATMENT
tipd=dat2$tipd                                      #TREATMENT (IPD VERSION)
mx=mean(x)                                          #AVERAGE COVARIATE VALUE
z=c(1,2,3,4,5, mx,0)                                #CHOSEN COVARIATE VALUES AT WHICH TREATMENT
EFFECTS  ARE REQUIRED TO BE ESTIMATED
nz=length(z)                                        #NUMBER OF CHOSEN COVARIATE VALUES
```

```
ns=max(s)                                  #NUMBER OF TRIALS
nt=max(tipd)                               #NUMBER OF TREATMENTS
np=length(y)                               #NUMBER OF PATIENTS

#LIST DATA FOR ENTRY INTO WINBUGS
data= list("y", "s","arm", "tipd", "b", "x", "z", "mx", "t", "na", "ns","nt", "np", "nz")

#DEFINE INITIAL VALUES FOR ENTRY INTO WINBUGS
inits1 = list(d=c(NA,0,0,0), sd=1, mu=c(0,0,0,0,0,  0,0,0,0,0,  0,0,0,0,0,  0,0), beta0=c(0,0,0,0,0,  0,0,0,0,0,  0,0,0,0,0,  0,0),
beta=c(NA,0,0,0))

#WINBUGS MODEL
Models1 = bugs (data, inits1, model.file= "NMA RE IPD COVM1.odc",
parameters.to.save= c("mu", "d",  "totresdev", "or", "lor", "sd", "tausq", "dz", "betas", "beta", "orz", "lorz", "beta0"),
n.chains=1, n.iter=300000, n.burnin=100000, n.thin=5,codaPkg=FALSE, bugs.directory="c:/Program Files/WinBUGS14/",
working.directory=working.directory)
```

Code for model S2

Winbugs code (saved as winbugs file "NMA RE IPD DSPLIT BETASPLIT.odc")

```
model{
for(i in 1:ns){                                  #LOOP FOR EACH TRIAL
   w[i,1] <- 0                                   #W IS ZERO FOR ARM 1 OF EACH TRIAL
     j[i,1] <-0                                  #J IS ZERO FOR ARM 1 OF EACH TRIAL
    delta[i,bi[i]] <- 0                          #TREATMENT EFFECT IS ZERO FOR ARM 1 OF EACH TRIAL
   mu[i] ~ dnorm(0,0.00001)                      #PRIOR DISTRIBUTION FOR MU
   beta0[i] ~ dnorm(0,0.00001)                   #PRIOR DISTRIBUTION FOR BETA0
            for (k in 1:na[i]) {                 #LOOP FOR EACH ARM
                index[i,k] <- split[i] * (equals(t[i,k], pair[1]) + equals(t[i,k], pair[2]))  #INDICATES IF ARM IS TO BE SPLIT
                              }
        for (k in 2:na[i]) {
             delta[i,si[i,k]] ~ dnorm(md[i,si[i,k]],taud[i,si[i,k]])          #DISTRIBUTION OF TRIAL-
                                                                              SPECIFIC TREATMENT EFFECTS
             md[i,si[i,k]] <-  (d[si[i,k]] - d[bi[i]] + sw[i,k])*(1-index[i,m[i,k]]) + direct*index[i,m[i,k]]
                                                                              #MEAN OF DISTRIBUTION
                                                                               (CORRECTED FOR MULTI-ARM
                                                                               TRIALS) SPLIT INTO DIRECT AND
                                                                               INDIRECT
               j[i,k] <- k - (equals(1, split[i]) * step(k-3))
             taud[i,si[i,k]] <- tau *2*(j[i,k]-1)/j[i,k]                      #PRECISION OF DISTRIBUTION
                                                                              (CORRECTED FOR MULTI-ARM
                                                                              TRIALS)

             w[i,k] <- (delta[i,si[i,k]]  - d[si[i,k]] + d[bi[i]]) * (1-index[i,k])     #ADJUSTMENTS FOR MULTI-ARM
                                                                               TRIALS
             sw[i,k] <- sum(w[i,1:k-1])/(j[i,k]-1)                            #ADJUSTMENTS FOR MULTI-ARM
                                                                              TRIALS
                            }
               }

for(l in 1:np)  {                                                             #LOOP FOR EACH PATIENT
  y[l]~dbern(p[l])                                                            #BERNOULLI LIKELIHOOD
     logit(p[l])<-mu[s[l]]  +  beta0[s[l]]*(x[l]-mx)  +  delta[s[l], tipd[l]] + (deltab[l]*(1-equals(tipd[l],bi[s[l]])) )
                                                                              #LINEAR PREDICTOR
  rhat[l] <- p[l]                                                             #MODEL PREDICTION
  dev[l] <- 2*(y[l] * (log(y[l]/rhat[l])) + (1-y[l]) * (log((1-y[l])/(1-rhat[l]))))    #DEVIANCE
     index2[l] < - split[s[l]] *  (equals(tipd[l], pair[1]) + equals(tipd[l], pair[2]))     #INDICATES IF ARM IS TO BE SPLIT
     deltab[l] <-  (beta[tipd[l]] - beta[bi[s[l]]] )*(x[l]-mx)*(1-index2[l]) + directbeta*(x[l]-mx)*(index2[l])
                                                                              #TREATMENT BY COVARIATE
                                                                                INTERACTION TERM SPLIT INTO
                                                                                DIRECT AND INDIRECT
                }

totresdev <- sum(dev[])                          #TOTAL RESIDUAL DEVIANCE

direct ~ dnorm(0,0.00001)                        #PRIOR DISTRIBUTION OF LOG ODDS RATIO FROM
                                                   DIRECT EVIDENCE
directbeta ~ dnorm(0,0.00001)                    #PRIOR DISTRIBUTION OF COEFFICIENT FROM DIRECT
                                                   EVIDENCE
d[1]<-0                                          #LOG ODDS RATIO IS ZERO FOR REFERENT TREATMENT
beta[1] <- 0                                     #COEFFICIENT IS ZERO FOR REFERENT TREATMENT

sd ~ dunif(0,10)                                 #PRIOR DISTRBIUTION FOR BETWEEN TRIAL STANDARD
                                                   DEVIATION
tau <- pow(sd,-2)                                #BETWEEN TRIAL PRECISION
tausq <- sd*sd                                   #BETWEEN TRIAL VARIANCE

for (k in 2:nt){
   d[k] ~ dnorm(0,0.00001)                       #PRIOR DISTRIBUTIONS FOR LOG ODDS RATIO  AND
                                                   COEFFICIENT FROM INDIRECT EVIDENCE
   beta[k]~dnorm(0,0.00001)
               }

for (k in 1:nt){                                 #CALCULATE THE LOG ODDS RATIO FOR BASIC
```

```
                                                    PARAMETERS AT EACH COVARIATE VALUE FOR
                                                    INDIRECT EVIDENCE

    for (v in 1:nz) { dz[v,k] <- d[k] - (beta[k])*(mx-z[v]) }
           }

for (c in 1:(nt-1)){                                #CALCULATE, FOR EACH COMPARISON, THE
                                                     COEFFICIENT, ODDS RATIO AND LOG ODDS RATIO AT
                                                     MEAN COVARIATE VALUE FOR INDIRECT EVIDENCE.
   for (k in (c+1):nt)  {
        betas[c,k] <- beta[k] - beta[c]
        lor[c,k] <- (d[k]-d[c])
              for (v in 1:nz) {
                                                    #CALCULATE, FOR EACH COMPARISON, ODDS RATIO AND
                                                     LOG ODDS RATIO AT DIFFERENT COVARIATE VALUES
                                                     FOR INDIRECT EVIDENCE.
        lorz[v,c,k] <- (dz[v,k]-dz[v,c])
                              }
                        }
              }

for (v in 1:nz) {                                   #LOG ODDS RATIO  AND ODDS RATIO AT EACH
                                                     COVARIATE VALUE FOR DIRECT EVIDENCE.

      directz[v] <- direct - (directbeta)*(mx-z[v])
      directorz[v] <-exp(directz[v])
              }

for (v in 1:nz) {
      diff[v] <- directz[v] - lorz[v, pair[1], pair[2]]      #CALCULATE INCONSISTENCY ESTIMATES
      prob[v] <- step(diff[v])                              #CALCULATE P-VALUES
                }
}
```

R code

```
#INSTALL R PACKAGES
library(R2WinBUGS)
library(coda)

#CHOOSE WORKING DIRECTORY
working.directory="c:\\dir"
setwd(working.directory)

#LOAD FUNCTIONS TO SHAPE DATA

#CHECK IF PAIR(X,Y) IN ROW I OF DATA AND GIVE BASELINE FOR DATA ROW I
PairXY <- function(treat, pair)
{
 N <- nrow(treat)
 out <- cbind(split=rep(0,N), b=rep(0,N))
 for (i in 1:N) {
  pos <- match(pair, treat[i,], nomatch=0)   # lenght = length(pair) = 2
  out[i,1] <- ifelse(prod(pos)>0, 1, 0)      # 1 if pair in line i, 0 o.w.
  out[i,2] <- ifelse(prod(pos)==0, 1, pos[1])
 }
out
}

# GIVES NA-1 INDEXES TO SWEEP NON-BASELINE ARMS ONLY
NonbaseSweep <- function(index, na)
{
 N <- NROW(na)
 C <- max(na)
 out <- matrix(nrow=N, ncol=C)
 for (i in 1:N) {
  for (k in 2:na[i]) {
    out[i,k] <- k - (index[i,"b"] >= k)
```

```
   }
  }
out
}

# BUILDS MATRIX WITH NON-BASELINE TREATMENTS
Sweeptreat <- function(treat, m)
{
  N <- NROW(treat)
  C <- NCOL(m)
  out <- matrix(nrow=N, ncol=C)
  for (i in 1:N) {
   for (k in 2:C) {
     out[i,k] <- treat[i,m[i,k]]
   }
  }
out
}

## BUILDS VECTOR WITH BASELINE TREATMENTS
Basetreat <- function(treat, b)
{
  N <- nrow(treat)
  out <- rep(0,N)
  for (i in 1:N) {
   out[i] <- treat[i,b[i]]
  }
out
}

#IMPORT DATA
dat1 = read.csv("utf_ipdacc.csv")
dat2 = read.csv("utf_ipdacc2.csv")

#DEFINE VARIABLES THAT NEED TO BE ENTERED INTO THE WINBUGS MODEL
na=dat1$na                                              #NUMBER OF ARMS IN EACH STUDY
t=cbind(dat1$t1,dat1$t2,dat1$t3, deparse.level = 0)     #TREATMENT NUMBER
s=dat2$s                                                #STUDY NUMBER
y=dat2$y                                                #OUTCOME
tipd=dat2$tipd                                          #TREATMENT NUMBER
x=dat2$age/12                                           #COVARIATE VALUES
ns=max(s)                                               #NMUBER OF TRIALS
nt=max(tipd)                                            #NUMBER OF TREATMENTS
np=length(y)                                            #NUMBER OF PATIENTS
mx=mean(x)                                              #AVERAGE COVARIATE VALUE
z=c(1,2,3,4,5, mx,0)                                    #CHOSEN COVARIATE VALUES AT WHICH TREATMENT
EFFECTS  ARE REQUIRED TO BE ESTIMATED.
nz=length(z)                                            #NUMBER OF CHOSEN COVARIATE VALUES

#DEFINE INITIAL VALUES FOR ENTRY INTO WINBUGS
inits1 = list(direct=0, d=c(NA,0,0,0), mu=rep(0,ns), directbeta=0, beta0=c(0,0,0,0,0,  0,0,0,0,0,  0,0,0,0,0,  0,0),
beta=c(NA,0,0,0), beta=c(NA,0,0,0), sd=1)

#CHOOSE NODE TO SPLIT
pair <- c(2,3)

# CALCULATE SPLIT (=1 IF NODE TO SPLIT IS PRESENT AND 0 OTHERWISE)
checkPair <- PairXY(t, pair)

# BUILD VECTOR BI[I] WITH BASELINE TREATMENT: T[I, B[I]]
bi <- Basetreat(t, checkPair[,"b"])

# INDEXES TO SWEEP NON-BASELINE ARMS ONLY
m <- NonbaseSweep(checkPair, na)

# BUILD MATRIX SI[I,K] WITH NON-BASELINE TREATMENTS: T[I, M[I,K]]
si <- Sweeptreat(t,m)
```

```
#LIST DATA FOR ENTRY INTO WINBUGS
bugs.data(list("y"=y,"s"=s,"tipd"=tipd,
		"na" = na, "nt" = nt, "ns" = ns,"np" = np, "t"=t,
		"split" = checkPair[,"split"], "m" =m,
		"bi" = bi, "si" = si, "pair" = pair, "x"=x, "z"=z, "nz"=nz,"mx"=mx ) )

#WINBUGS MODEL
modelS2=bugs(data = "data.txt",
    inits = inits1, parameters.to.save = c("direct", "d", "lor", "mu", "prob","totresdev", "diff", "directbeta", "directz", "lorz", "betas", "dz","beta", "sd", "tausq"), model.file = "NMA RE IPD DSPLIT BETASPLIT.odc",
    n.chains = 1,  n.iter = 300000, n.burnin = 100000,  bugs.directory = "C:/Program Files/WinBUGS14/", working.directory=working.directory)

######REPEAT FOR OTHER NODES
```

Site	Artemisinin-based combination therapies (number of patients that achieved treatment success/number of patients)				Age in years, mean (standard deviation)
	**DHAPQ**	**AQ+AS**	**AL**	**CD+A**	
Manhica (after CD+A)	94/100	78/97	-	-	2.88 (1.30)
Mbarara (after CD+A)	63/65	59/70	-	-	2.43 (1.07)
Nanoro	187/219	199/290	115/292	-	2.24 (1.18)
Gabon	62/63	67/76	65/70	-	2.83 (1.28)
Afokang	67/72	78/83	84/87	-	2.94 (1.28)
Pamol	60/65	73/79	73/80	-	2.66 (1.36)
Ndola	67/67	63/69	63/75	-	2.45 (1.20)
Manhica (before CD+A)	78/82	70/86	-	42/84	2.82 (1,00)
Mbarara (before CD+A)	72/80	64/79	-	53/80	2.60 (1.10)
Rukara (after CD+A)	46/47	-	46/50	-	3.08 (0.92)
Jinja (after CD+A)	160/167	-	157/168	-	2.33 (1.17)
Tororo (after CD+A)	54/75	-	33/77	-	1.99 (0.99)
Mashesha (after CD+A)	49/52	-	51/52	-	2.90 (1.05)
Rukara (before CD+A)	22/23	-	18/21	4/23	2.71 (1.00)
Jinja (before CD+A)	37/39	-	35/38	34/40	2.62 (1.19)
Tororo (before CD+A)	109/141	-	88/138	71/142	2.11 (0.85)
Mashesha (before CD+A)	23/24	-	23/23	18/24	2.92 (1.09)

Table S1. Summary of the individual patient data (i.e. event rate of each treatment group of each site for treatment success at day 28) and covariate information.
AQ+AS: amodiaquine-artesunate; AL: artemether-lumefantrine; CD+A: chlorproguanil-dapsone plus artesunate; DHAPQ: dihydroartemisinin-piperaquine.

Comparison	Evidence type	Odds ratio<br>Posterior median (posterior 95% credibility interval)					
		Age 1	Age 2	Mean age i.e. 2.5	Age 3	Age 4	Age 5
**AL vs. AQ+AS**	**Direct**	0.65 (0.26, 1.76)	0.71 (0.29, 1.81)	0.74 (0.31, 1.87)	0.77 (0.31, 1.96)	0.83 (0.32, 2.26)	0.90 (0.31, 2.72)
	**Indirect**	2.65 (0.86, 9.44)	1.89 (0.72, 5.88)	1.60 (0.61, 4.90)	1.36 (0.50, 4.26)	0.98 (0.29, 3.58)	0.71 (0.15, 3.37)
**CD+A vs. AQ+AS**	**Direct**	0.66 (0.13, 3.47)	0.43 (0.10, 1.92)	0.34 (0.08, 1.50)	0.28 (0.06, 1.21)	0.18 (0.04, 0.87)	0.11 (0.02, 0.70)
	**Indirect**	0.26 (0.06, 1.02)	0.23 (0.06, 0.80)	0.22 (0.06, 0.75)	0.21 (0.06, 0.75)	0.19 (0.04, 0.83)	0.17 (0.03, 1.01)
**CD+A vs. AL**	**Direct**	0.24 (0.06, 0.82)	0.21 (0.06, 0.62)	0.20 (0.06, 0.58)	0.18 (0.05, 0.56)	0.16 (0.04, 0.59)	0.14 (0.03, 0.70)
	**Indirect**	0.69 (0.13, 3.25)	0.43 (0.10, 1.68)	0.34 (0.08, 1.30)	0.26 (0.06, 1.04)	0.16 (0.03, 0.75)	0.10 (0.02, 0.62)

Table S2. Odds ratios for treatment success from the NMA node-splitting models including interactions (*model S2*).
AQ+AS: amodiaquine-artesunate; AL: artemether-lumefantrine; CD+A: chlorproguanil-dapsone plus artesunate; DHAPQ: dihydroartemisinin-piperaquine.

**Comparison**	**Log odds ratio<br>Posterior median (posterior 95% credibility interval)**					
	**Age 1**	**Age 2**	**Mean age<br>i.e. 2.5**	**Age 3**	**Age 4**	**Age 5**
**AL vs. AQ+AS**	0.05<br>(-0.74, 0.92)	0.05<br>(-0.67, 0.87)	0.06<br>(-0.67, 0.87)	0.06<br>-0.68, 0.88	0.07<br>-0.75, 0.94	0.08<br>-0.86, 1.05
**CD+A vs. AQ+AS**	-0.93<br>(-2.02, 0.11)	-1.20<br>(-2.18, -0.27)	-1.34<br>(-2.30, -0.42)	-1.47<br>-2.45, -0.54	-1.74<br>-2.83, -0.70	-2.02<br>-3.30, -0.77
**CD+A vs. AL**	-0.98<br>(-2.07, -0.01)	-1.25<br>(-2.24, -0.41)	-1.39<br>(-2.36, -0.56)	-1.53<br>-2.51, -0.68	-1.82<br>-2.89, -0.82	-2.10<br>-3.37, -0.88

Table S3. Selected results for treatment success from the NMA model including interactions (*model S1*).
AQ+AS: amodiaquine-artesunate; AL: artemether-lumefantrine; CD+A: chlorproguanil-dapsone plus artesunate; DHAPQ: dihydroartemisinin-piperaquine. The between trial variance was 0.77 (0.27, 2.07).

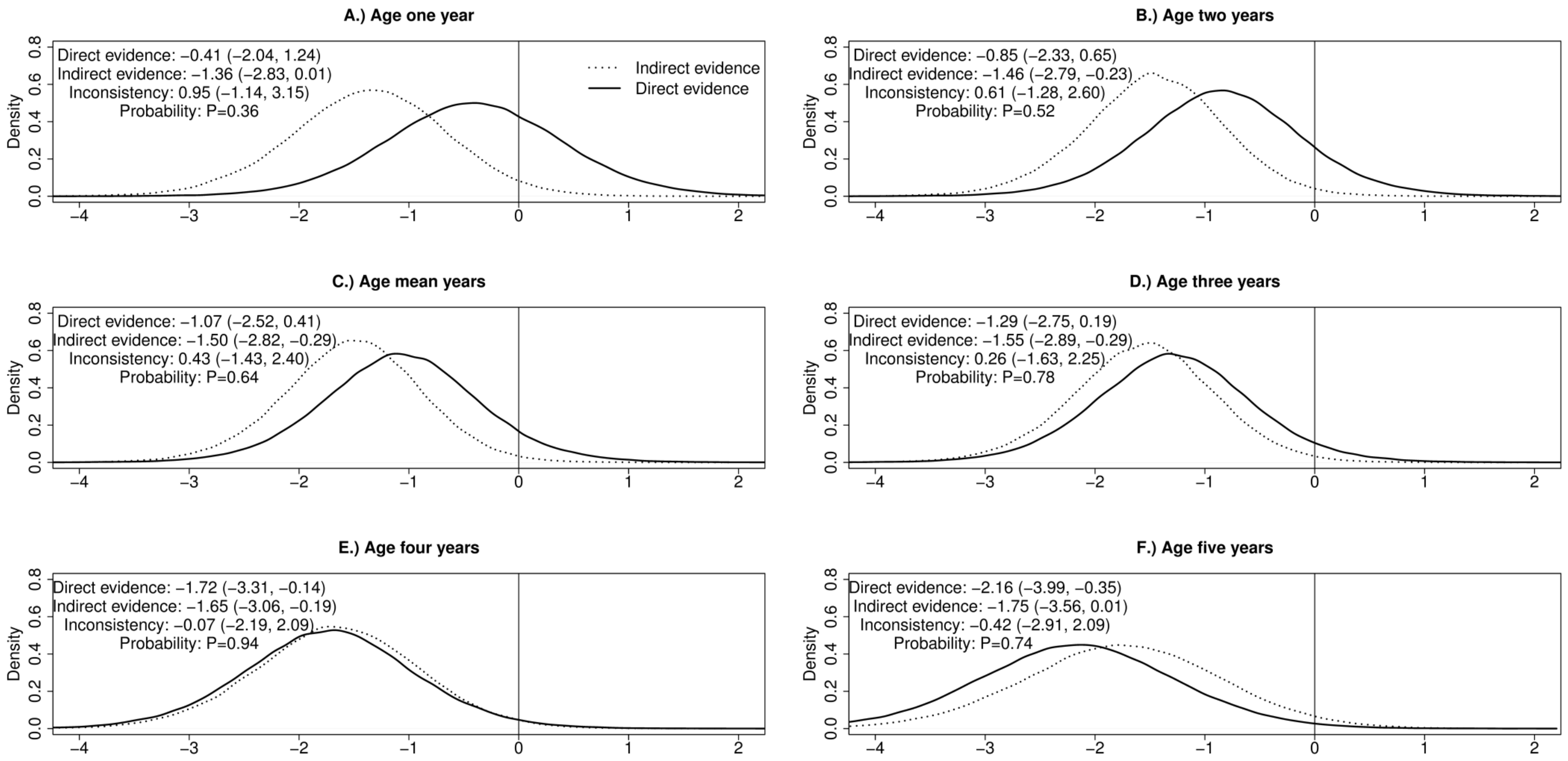

Figure S1. Posterior distributions of log odds ratios at various ages for treatment success for CD+A versus AQ+AS.

The mean age was 2.5 years.

AQ+AS: amodiaquine-artesunate; CD+A: chlorproguanil-dapsone plus artesunate.

Posterior median (95% credibility interval) presented.

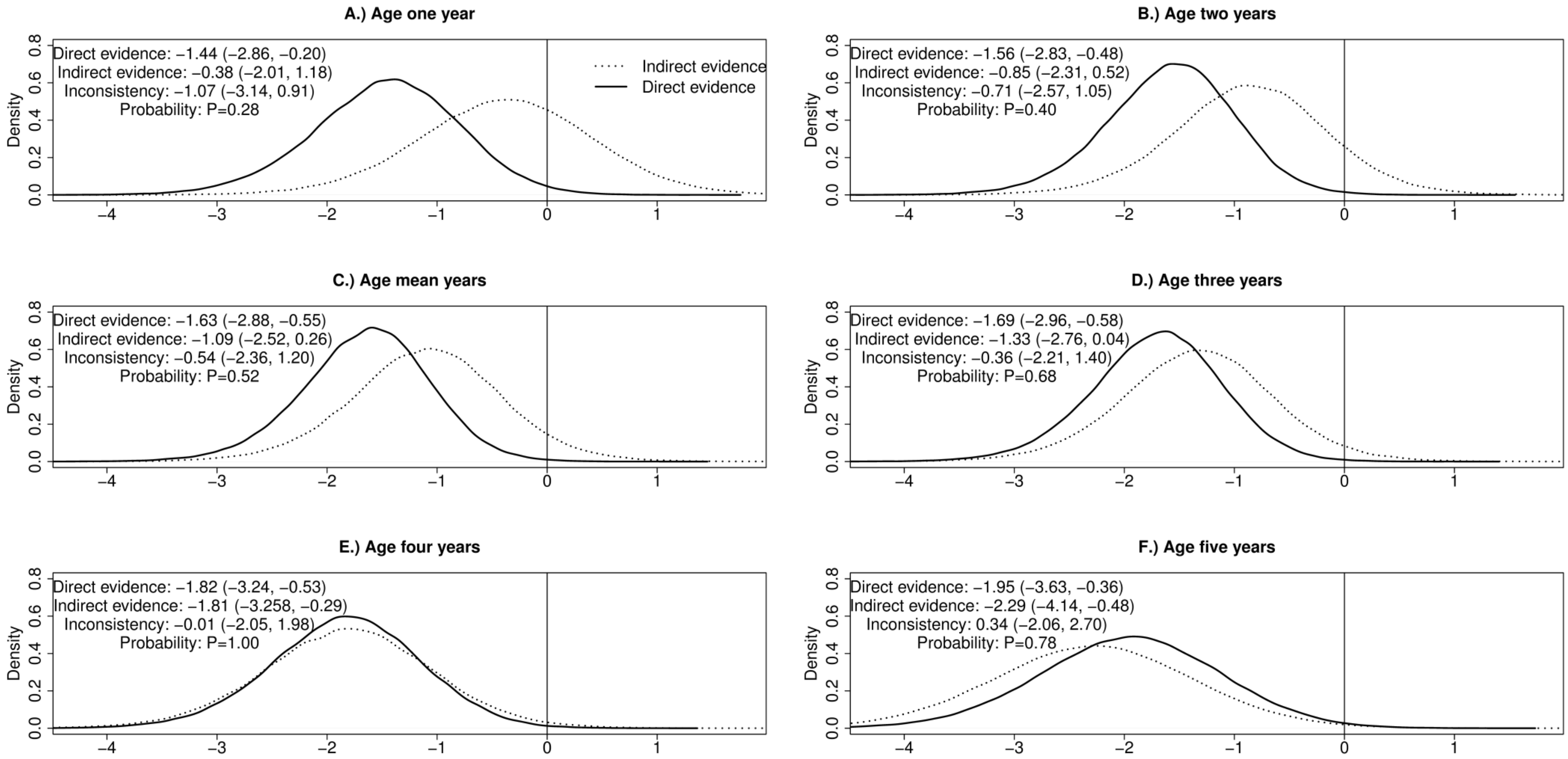

Figure S2. Posterior distributions of log odds ratios at various ages for treatment success for CD+A versus AL.

The mean age was 2.5 years.

AL: artemether-lumefantrine; CD+A: chlorproguanil-dapsone plus artesunate.

Posterior median (95% credibility interval) presented.
